# The mediating role of fragility of happiness in the association between marital loneliness and depression sensitivity: an actor-partner interdependence analysis

**DOI:** 10.3389/fpsyg.2026.1870144

**Published:** 2026-07-01

**Authors:** Süleyman Akçıl, Beste Erdinç

**Affiliations:** 1 Ereğli Faculty of Education, Zonguldak Bülent Ecevit University, Ereğli, Zonguldak, Türkiye; 2 İstanbul Nişantaşı University, İstanbul, Türkiye

**Keywords:** actor-partner interdependence model, depression sensitivity, fragility of happiness, loneliness, married couples

## Abstract

**Introduction:**

Although marriage generally supports psychological wellbeing, individuals can still experience profound loneliness within intimate relationships, which may increase susceptibility to adverse mental health outcomes. This study employed a cross-sectional, dyadic research design to investigate whether the belief that happiness is fragile mediates the relationship between marital loneliness and depression sensitivity.

**Methods:**

Self-report data were collected from 307 married couples (*N* = 614; husbands’ mean age = 35.71, wives’ mean age = 33.24) in Türkiye. Both intrapersonal (actor) and interpersonal (partner) pathways were examined by adopting the Actor-Partner Interdependence Mediation Model (APIMeM) via structural equation modeling.

**Results:**

Results revealed significant actor and partner mediation effects for both husbands and wives (e.g., actor effect: β = 0.39, *p* < 0.01; partner effect: β = 0.27, *p* < 0.01). Specifically, an individual’s marital loneliness predicted their own depression sensitivity through their personal belief that happiness is fragile (actor effect). Furthermore, cross-partner effects showed that one spouse’s loneliness predicted the other’s depression sensitivity through the partner’s heightened perception of fragile happiness.

**Discussion:**

These findings illustrate how cognitive appraisals of happiness transmit relational loneliness into depressive vulnerabilities. Addressing maladaptive beliefs about the fragility of happiness in couple therapy may help prevent marital loneliness from exacerbating depressive symptoms for both partners.

## 1 Introduction

The majority of people look for methods to be fulfilled in all facets of their lives (Diener, 2000). A lasting source of happiness for people might be starting a family and maintaining a happy marriage relationship. The most effective strategy to increase happiness is not to change people’s life circumstances (e.g., marital status, career, location, and income) (Sheldon and Lyubomirsky, 2006). Beyond merely altering living circumstances, however, strong social bonds, like marriage, can be crucial in providing for a person’s basic emotional requirements. For instance, income is not as good a predictor of daily positive and negative emotions as addressing one’s basic psychological needs—namely, autonomy, competence, and relatedness (Ryan and Deci, 2000; Diener et al., 2010). However, depression is a prevalent mental health concern that profoundly impacts these marital dynamics (Beach et al., 1990; Whisman, 2001). While marriage is generally considered a protective factor for psychological wellbeing, distressed marriages can serve as a significant source of chronic stress, increasing susceptibility to depressive symptoms (Bulanda et al., 2024; Proulx et al., 2007). Indeed, dyadic longitudinal research confirms a strong, bidirectional sequence between relationship dissatisfaction and depressive symptoms among partners over time (Morgan et al., 2018). According to the marital discord model of depression, problematic relationship dynamics directly contribute to an individual‘s depressive vulnerability (Beach et al., 1990; Fincham et al., 1997). In the context of intimate relationships, depression sensitivity refers to the internal anxieties and vulnerabilities individuals develop in response to the threat of depressive symptoms (Capron et al., 2021). Spouses who experience severe relational distress may become more sensitive to emotional fluctuations, perceiving them as hazardous to their overall wellbeing.

A critical element of relational distress that exacerbates depressive symptoms is marital loneliness. Because it satisfies the desire for relatedness, a successful married relationship can now support happy feelings more than other socioeconomic aspects. In fact, within a dyadic context, satisfying this fundamental need for relatedness not only serves as a crucial predictor of romantic relationship quality, but its absence also significantly increases loneliness (Akyıl et al., 2026). This subjective sense of isolation, even when married, poses a severe risk to mental and physical health (Hawkley and Cacioppo, 2010). Grounded in the Stress Generation Theory (Hammen, 1991), relational stressors such as marital loneliness not only arise from individual vulnerabilities but also actively generate further psychological distress, creating a cyclical relationship with depression (Davila et al., 1997). Supporting this, recent evidence highlights that marital loneliness often serves as a key mechanism linking relationship distress to heightened depressive vulnerabilities (Marini et al., 2020). Marital loneliness can be especially upsetting because it deviates from marital standards and can negatively affect a person’s physical, emotional, and spiritual wellbeing (Rokach and Sha’ked, 2013).

To understand the cognitive mechanisms translating marital loneliness into depression sensitivity, it is essential to examine specific cognitive schemas such as the fragility of happiness. Happiness is a collection of individual ideas and conceptions that could have significant real-world repercussions (Joshanloo, 2019). Many people and cultures hold the view that happiness is fragile, or ephemeral, and can quickly devolve into less favorable circumstances (Joshanloo et al., 2015). Those who place an excessive priority on happiness are also less likely to achieve long-term happiness (Luhmann et al., 2016). People generally believe that they are susceptible to mental health issues. Overvaluing happiness has been linked to increased depression symptoms in a number of studies (Bardeen and Fergus, 2020; Fergus and Bardeen, 2016). Valuing happiness is known to be linked to negative outcomes, including loneliness (Mauss et al., 2012) and depression symptoms (Ford et al., 2015). It also has a substantial positive relationship with the fragility of happiness (Joshanloo, 2018). Thus, the cognitive appraisal that happiness is fragile is hypothesized to act as a crucial mediating variable that links the experience of marital loneliness to increased depression sensitivity.

Understanding the relationship between marital dynamics, loneliness, and mental health requires a focus on the interdependent nature of romantic relationships. Studying the actor-partner effects of happiness in this context is highly appropriate and important because spouses’ emotional states and cognitive appraisals are deeply interconnected. Recent longitudinal studies underscore this interdependence, highlighting that trajectories of marital quality, loneliness, and social isolation are robust and bidirectional predictors of depressive symptoms and loneliness over time (Bloomberg et al., 2025; Hsu et al., 2023; Tucker et al., 2021; Wickrama et al., 2020). These longitudinal designs reveal that marital strain dynamically influences both spouses’ psychological wellbeing (Joosten et al., 2022). Therefore, relying on advanced analytical approaches like the Actor-Partner Interdependence Model (APIM) is critical. Analyzing marital dyads requires a specialized statistical framework that can account for the mutual influence between spouses. Employing an APIM allows for the simultaneous estimation of how an individual‘s emotional state affects their own wellbeing (actor effect) as well as their partner’s wellbeing (partner effect) (Abaei and Martin, 2023). Establishing the dyadic nature of these variables is a key contribution of this study, because recent longitudinal evidence demonstrates that happiness among adults is much more strongly tied to close, intimate relationships with their spouses compared to their relationships with children or friends (Abaei and Martin, 2026). Thus, an APIM approach provides the most appropriate and conceptually sound analytical framework to capture how emotional distress is transmitted within the dyad.

### 1.1 Present study

Grounded in the framework of Family Systems Theory (Bowen, 1978) and Emotional Contagion Theory (Hatfield et al., 1993), which characterize a marital dyad as a highly interdependent emotional unit, cognitive schemas and emotional vulnerabilities are not isolated within the individual but are continuously transmitted and shared between spouses. This theoretical framework helps clarify how personal psychological distress affects the broader relationship. Most people see happiness as a key component of a good life (King and Napa, 1998). Although marital relationships are fundamentally expected to provide sustained social support, the experience of loneliness within a marriage represents a profound relational distress. From the perspective of Family Systems Theory, one partner’s loneliness affects the emotional balance of the entire dyad.

The current study intends to investigate the relationship between marital loneliness and depression sensitivity, as well as the mediating effect of fragility of happiness. A dyadic analysis of married couples is carried out for this reason. We propose that the cognitive belief that “happiness is fragile” acts as a critical transmission mechanism (mediator) within the marital system. This study addresses significant gaps in the literature by offering theoretical, methodological, and practical contributions. Theoretically, it integrates individual cognitive appraisals (fragility of happiness) with relational distress (loneliness) within a systemic framework. Methodologically, utilizing the Actor-Partner Interdependence Mediation Model (APIMeM) allows for a rigorous, simultaneous examination of both intrapersonal and interpersonal pathways. Practically, understanding these mechanisms provides crucial insights for couple therapy, highlighting the need to target maladaptive systemic beliefs about happiness to prevent the mutual escalation of depressive symptoms.

Given the central role of close relationships in most individuals’ lives, the mental health and emotional experiences of both partners mutually influence the overall functioning of the relationship (Akçıl, 2026). Accordingly, to specifically capture the distinct intrapersonal and interpersonal pathways within our distinguishable dyad framework, the following four hypotheses were formulated:

1. H1 (Actor effect for women): Wives’ belief in the fragility of happiness significantly mediates the association between their own marital loneliness and their own depression sensitivity.
2. H2 (Actor effect for men): Husbands’ belief in the fragility of happiness significantly mediates the association between their own marital loneliness and their own depression sensitivity.
3. H3 (Partner effect from women to men): Husbands’ belief in the fragility of happiness significantly mediates the association between wives’ marital loneliness and husbands’ depression sensitivity.
4. H4 (Partner effect from men to women): Wives’ belief in the fragility of happiness significantly mediates the association between husbands’ marital loneliness and wives’ depression sensitivity.

## 2 Materials and methods

### 2.1 Participants

A cross-sectional, dyadic research design was used for this study. The target population was married individuals living in Türkiye. We used a convenience sampling method through social media platforms to recruit volunteer participants. To ensure a homogenous sample, couples were included if they: (a) were legally married and living in the same household, (b) were at least 18 years old, (c) resided in Türkiye, and (d) volunteered to participate. We excluded couples if they did not meet these criteria or were going through a divorce or separation.

The final sample consisted of 307 heterosexual married couples (*N* = 614 individuals). When determining the required sample size for Structural Equation Modeling (SEM) and APIM analyses, methodological guidelines strongly recommend a minimum of 200 cases, as well as satisfying an adequate case-to-parameter ratio to achieve adequate statistical power and parameter stability (Kline, 2016). Thus, our sample size of 307 dyads comfortably exceeded this recommended threshold, providing robust statistical power to test the hypothesized complex mediation pathways.

Regarding the demographic profile of the participants, the men were, on average, 35.71 years old (SD = 8.76) and the women were 33.24 years old (SD = 1.17). Regarding marriage length, 31.6% of the couples had been married for 10 years or more. Although 37.8% of the couples did not have children, the rest had at least one child. Most participants had a university degree (66.8% of men and 65.1% of women).

### 2.2 Materials

#### 2.2.1 Fragility of happiness scale

Developed by Joshanloo et al. (2015), this unidimensional scale measures the belief that happiness is fleeting and easily lost. In 2021, Yıldırım and Çelik Tanrıverdi (2021) validated it in Turkish. The scale consists of four items evaluated on a seven-point Likert-type scale ranging from 1 (*strongly disagree*) to 7 (*strongly agree*). The total score ranges from 4 to 28, with higher scores indicating a stronger belief in the fragility of happiness. A sample item from the scale is: “Something might happen at any time and we could easily lose our happiness.” The Turkish version of the scale demonstrated appropriate fit indices, according to the CFA results [χ^2^ (2) = 4.84, *p* = 0.09, CIMIN/DF = 2.42, NFI = 0.97, CFI = 0.99, and RMSEA = 0.08]. The internal consistency reliability (Cronbach‘s α) was reported as 0.84 in the adaptation study. In the current study, the internal consistency reliability was 0.80 for women and 0.83 for men.

#### 2.2.2 Loneliness in intimate relationships scale

The scale was created by Rokach et al. (2022) to evaluate the loneliness component of intimate relationships. It was validated in Turkish by Ekşi and Kısrık (2023). The scale has fourteen items and three subscales (separation, hurt, and guilt). Responses are rated on a six-point Likert scale (1 = totally not describes my situation to 6 = totally describes my situation). The scale results in a minimum score of 14 and a maximum score of 84. In intimate relationships, a high score on the scale denotes a significant degree of loneliness. Sample items for each subscale include: “My spouse/partner had no time for me” (separation), “I felt insulted” (hurt), and “I felt guilty for my misdeeds in the marriage/relationship” (guilt). The Turkish adaptation demonstrated acceptable fit values (χ2/df = 3.03, RMSEA = 0.074, CFI = 0.912, TLI = 0.889, SRMR = 0.04) and a reliability coefficient of 0.85. In the current study, Cronbach‘s α for the overall scale was 0.86 for both women and men.

#### 2.2.3 Depression sensitivity index

Çıtak et al. (2024) validated this scale, which was created by Capron et al. (2021) and utilized in adult samples, into Turkish. Physical/cognitive issues and social concerns are the two subscales of the scale. Cronbach‘s alpha internal consistency coefficient for the entire scale was determined to be 0.82 in the scale‘s development research. The scale comprises nine items with five-point Likert-type response possibilities that range from 1 (very little) to 5 (very much). The scale has a maximum possible score of 45 and a minimum possible value of 9. A higher score on the scale indicates a greater susceptibility to depressive symptoms. Sample items for each subscale include: “If I can’t sleep I worry that there is something wrong with me” (physical/cognitive concerns) and “It is important to me not to appear depressed” (social concerns). The Turkish adaptation yielded excellent model fit indices (χ2 = 88.64, df = 26, CFI = 0.963, TLI = 0.93, RMSEA = 0.066). Cronbach’s alpha internal consistency coefficient for the entire scale was determined to be 0.82 in the scale’s development research. In the present study, the internal consistency coefficient was 0.81 for women and 0.79 for men.

### 2.3 Procedure

The Yıldız Technical University Scientific Research and Ethical Review Board approved the study on 2 May 2025 (Report Number: 202505). Data collection began shortly after and continued until October 2025. We distributed the secure online survey link directly across various social media platforms. To reach a broader audience, we also asked participating couples to share the link with other eligible couples in their networks, utilizing a snowball sampling technique. Before starting the survey, all participants read an information sheet detailing the study’s purpose and provided informed consent electronically.

To ensure the data from each spouse remained independent, we gave each couple a unique identification code. We explicitly instructed spouses to complete the survey separately, at different times or on different devices, and asked them not to discuss their answers with each other. After the data collection phase ended, we used the unique identification codes to match the responses of husbands and wives. During the initial data screening process, prior to any statistical modeling, we identified and removed dyads who submitted incomplete questionnaires. Handling these incomplete responses during the data cleaning phase ensured the integrity of the final matched dataset, which was then prepared for dyadic analysis. The study followed the ethical guidelines outlined in the Declaration of Helsinki.

### 2.4 Data analysis

This study examined whether the fragility of happiness mediated the relationship between marital loneliness and depression sensitivity in couples. The study examined the direct and indirect effects of couples on one another and themselves using a reciprocal-relational model.

To create the final data collection, the information gathered from the pairings was first matched with pseudonyms. IBM SPSS Statistics 23 was used to do preliminary analyses of the data, such as reliability, descriptive, and correlational analysis (Table 1). The structural model was then tested using path analysis, and AMOS was utilized to apply dyadic analysis techniques. Item-parceling was applied to the measurement models. While the fragility of happiness was parceled as a unidimensional construct, the loneliness in intimate relationships scale inherently possesses a multidimensional structure. However, rather than modeling its subdimensions separately, the items of the loneliness scale were aggregated into three parcels to represent the overall loneliness construct. According to the literature, when the research goal is to evaluate a latent variable at a broader, general level rather than focusing on its specific subdimensions, parceling can effectively minimize lower-level nuisance factors and clarify the overall representation of multidimensional constructs (Little et al., 2002, 2013). Conversely, the predicted variable, depression sensitivity, was not parceled to observe its specific dimensions (physical/cognitive issues and social concerns) as distinct outcomes.

**TABLE 1:** Descriptive statistics and reliabilities for the study variables.

Variable	1	2	3	4	5	6
1. Loneliness (female)	–	–	–	–	–	–
2. Loneliness (male)	0.42**	–	–	–	–	–
3. Fragility of happiness (female)	0.27**	0.24**	–	–	–	–
4. Fragility of happiness (male)	0.29**	0.35**	0.35**	–	–	–
5. Depression sensitivity (female)	0.28**	0.14**	0.34**	0.23**	–	–
6. Depression Sensitivity (male)	0.16**	0.50**	0.24**	0.33**	0.20**	–
Mean	40.58	36.99	18.64	18.17	22.23	19.94
SD	13.38	12.80	5.69	5.96	7.07	6.60
Skewness	0.054	0.387	−0.398	−0.450	0.325	0.486
Kurtosis	−0.139	0.088	−0.513	−0.350	0.303	0.067
McDonald ω	0.853	0.864	0.809	0.832	0.814	0.788
Cronbach α	0.862	0.861	0.803	0.828	0.810	0.793
Guttmann λ6	0.895	0.891	0.772	0.800	0.843	0.825

**Indicates that the correlation is statistically significant at the *p* < 0.001 level.

In evaluating the overall fit of the models, several specific fit indices were considered, and cut-off values were determined based on standard criteria (Hu and Bentler, 1999). Specifically, a non-significant *x*^2^ value (*p* > 0.05) is traditionally desired, though it is sensitive to large sample sizes. Therefore, alternative fit indices were relied upon: a Comparative Fit Index (CFI) and a Tucker-Lewis Index (TLI) ≥ 0.90 indicate acceptable fit (with values ≥ 0.95 indicating excellent fit), and a Root Mean Square Error of Approximation (RMSEA) value ≤ 0.08 indicates acceptable fit (with values ≤ 0.05 indicating excellent fit). The Actor-Partner Interdependence Mediation Model (APIMeM), an extension of APIM, and one of the binary analysis techniques, APIM, was employed. To examine the significance of the indirect effects within the APIMeM framework, a bootstrapping procedure with 5,000 resamples was conducted in AMOS. Indirect effects were evaluated using bias-corrected 95% confidence intervals (CIs). Consistent with current recommendations for mediation analyses, an indirect effect was considered statistically significant when the corresponding 95% bootstrap confidence interval did not include zero.

## 3 Results

### 3.1 Descriptive statistics, gender differences, and correlations

Table 1 displays descriptive statistics for the variables examined within the parameters of the study, as well as their correlations. The examination of the actor correlations between the variables reveals a positive relationship between depression sensitivity (Female *r* = 0.28, *p* < 0.01; Male *r* = 0.50, *p* < 0.01) and loneliness as well as the fragility of happiness (Female *r* = 0.27, *p* < 0.01; Male *r* = 0.35, *p* < 0.01) for both men and women. Similarly, for both genders, fragility of happiness had a positive and significant relationship with depression sensitivity (Female: *r* = 0.34, *p* < 0.01; Male: *r* = 0.33, *p* < 0.01).

### 3.2 Actor-Partner Interdependence Mediation Model (APIMeM)

The measurement models that contained the spouses’ data, both independently and collectively, were tested first at this point. Three latent variables—loneliness, fragility of happiness, and depression sensitivity—and fourteen observable variables make up the measurement model. A good fit was found between the measurement models used to evaluate the data from men and women [for females: χ^2^ (11, *N* = 307) = 1.741; *p* > 0.05; TLI = 0.98; CFI = 0.99; RMSEA = 0.04; for males: χ^2^ (11, *N* = 307) = 2.457; *p* > 0.05; TLI = 0.96; CFI = 0.98; RMSEA = 0.066]. Lastly, a satisfactory fit was also demonstrated by the measurement model that was used to assess the data from both men and women combined; χ^2^ (62, *N* = 307) = 1.678, *p* > 0.05; TLI = 0.96; CFI = 0.97; RMSEA = 0.04.

**FIGURE 1:**
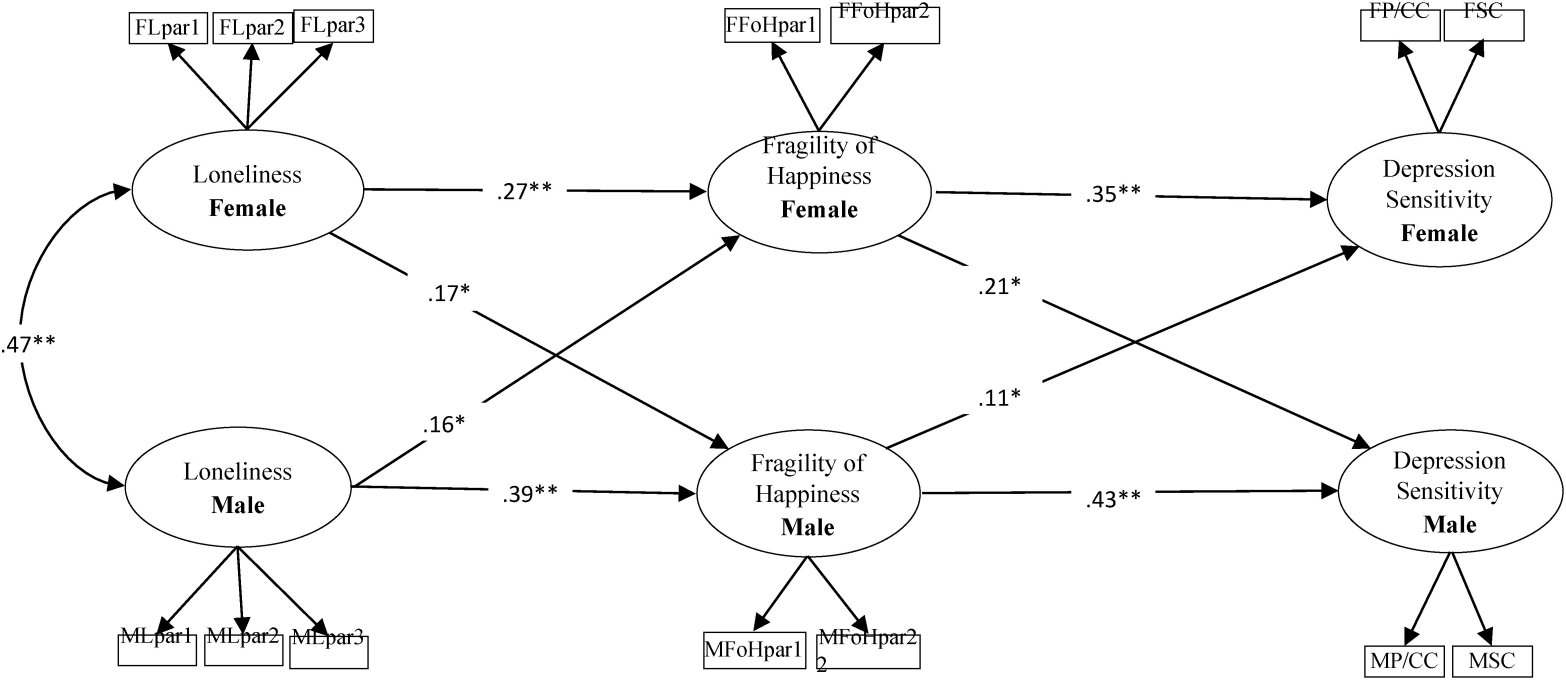
Actor-Partner Independence Mediation Model. *N* = 614;*p < 0.05, **p < 0.01; FLpar parcels of female’s loneliness; MLpar parcels of male’s loneliness; FFoHpar parcels of female’s fragility of happiness; MFoHpar parcels of male’s fragility of happiness.

The actor-partner interdependence mediation model was used to evaluate the mediation effect of fragility of happiness in the relationship between loneliness and depression sensitivity in couples after the measuring model. The model demonstrated acceptable fit indices: χ^2^/df = 2.756; *p* < 0.05; TLI = 0.91; CFI = 0.91; RMSEA = 0.07. Figure 1 depicts the structural model and standardized path coefficients, illustrating the mediating role of the fragility of happiness in the relationship between marital loneliness and depression sensitivity for both men and women. Both mediating actor-mediating partner effects and actor-partner effects are included in the model.

To further examine the significance of the actor and partner indirect effects, a bias-corrected bootstrap procedure with 5,000 resamples was conducted. The estimates of the indirect effects and their corresponding 95% bootstrap confidence intervals are presented in Table 2.

**TABLE 2:** Bootstrapping results.

Path	Coefficient	95% CI	
		LL	UL
Actor effects			
Loneliness (F) → fragility of happiness (F) → depression sensitivity (F)	0.113	0.044	0.209
Loneliness (M) → fragility of happiness (M) → depression sensitivity (M)	0.068	0.023	0.143
Partner effects			
Loneliness (F) → fragility of happiness (M) → depression sensitivity (F)	0.022	0.001	0.066
Loneliness (M) → fragility of happiness (F) → depression sensitivity (M)	0.014	0.001	0.051

F, female; M, male; CI, confidence interval; LL, lower limit; UL, upper limit.

As presented in Table 2, the bootstrap analyses generally supported the proposed actor and partner mediation pathways. However, given that the lower bounds of some confidence intervals were very close to zero, these indirect effects should be interpreted with caution.

## 4 Discussion

The primary purpose of this study was to investigate the mediating role of the fragility of happiness in the association between marital loneliness and depression sensitivity among married couples. Utilizing an Actor-Partner Interdependence Mediation Model (APIMeM), we examined both intrapersonal (actor) and interpersonal (partner) pathways to capture the complex emotional dynamics within the marital dyad. Overall, the findings of the current study demonstrated that both actor and partner mediation effects were statistically significant. Specifically, marital loneliness exacerbated depression sensitivity not only for the individual experiencing it but also for their spouse, and this transmission was significantly mediated by the belief that happiness is fragile.

Hypotheses 1 and 2, which predicted that wives’ and husbands’ belief in the fragility of happiness would significantly mediate the association between their own marital loneliness and their own depression sensitivity (actor effects), were fully supported. Although not all marriages offer the same level of protection against loneliness, marriage generally provides a buffering effect (Hsieh and Hawkley, 2018). This finding indicates that the nature of the relationship matters and that marriage by itself does not offer emotional security. Marital loneliness can be especially upsetting because it deviates from marital standards and can negatively affect a person’s physical, emotional, and spiritual wellbeing (Rokach and Sha’ked, 2013). According to Joshanloo et al. (2015), people may be more susceptible to negative emotions due to increased sensitivity to emotional swings if they believe that happiness is fragile, or ephemeral, and can rapidly transform into less favorable circumstances. Particularly those who suffer ongoing loneliness in intimate relationships and think that happiness is fragile may eventually come to view depressed symptoms as a threat and form a more sensitive mental defense against them. Consistent with prior longitudinal evidence suggesting that relational distress robustly predicts depressive symptoms over time (Tucker et al., 2021), our findings indicate that this heightened cognitive fragility serves as an internal mechanism making individuals far more sensitive to depression.

Similarly, Hypotheses 3 and 4, which proposed cross-partner mediation effects from wives to husbands and from husbands to wives, were also supported. The subjective sense of social isolation is a popular definition of loneliness (Hawkley and Cacioppo, 2010). However, loneliness and isolation are not the same thing; people who are in long-term relationships and participate in social networks can also suffer from feeling isolated. According to Cacioppo et al. (2009), Stokes (2017), loneliness is partially contagious, meaning that when one person feels lonely, their intimate companions may also feel lonely. Consequently, when one partner in a married pair experiences loneliness, it may eventually lead to the other partner experiencing loneliness as well, which could have a detrimental effect on the relationship’s overall satisfaction. Demonstrating this dyadic nature, Hsieh and Hawkley (2018) found that individuals experiencing marital dissatisfaction experience greater loneliness (actor effect), and those who are dissatisfied also contribute to their spouses’ loneliness (partner effect). To understand how this shared distress impacts the dyad, it is vital to explicitly study the actor-partner effects of happiness and emotional wellbeing. Prior longitudinal work shows that happiness among older adults is strongly related to close relationships with spouses, even more so than relationships with children or friends (Abaei and Martin, 2026). Viewed through the foundational lens of Family Systems Theory (Bowen, 1978) and Emotional Contagion Theory (Hatfield et al., 1993), our findings extend this literature by showing that contagious negative affective states ripple through the marital system. This emotional contagion directly alters the non-lonely partner’s cognitive stability, causing them to evaluate the shared happiness of the relationship as precarious and fragile, which subsequently increases their depression sensitivity. This cross-spousal emotional transmission is heavily supported by recent dyadic literature showing that partners’ emotional wellbeing and depressive trajectories are profoundly intertwined (Abaei and Martin, 2023; Bulanda et al., 2024).

From a clinical perspective, these dyadic findings suggest that interventions targeting depressive vulnerabilities must shift away from an exclusively individualistic focus. Given that actor and partner effects dynamically interact within the marital system, family and couple therapists should design interventions that go beyond individual cognitive restructuring. Therapists should actively address maladaptive systemic beliefs regarding the fragility and instability of positive emotions. By helping couples dismantle the cognitive perception that happiness is fundamentally fleeting and by reconstructing secure, responsive relational bonds, therapeutic practices can successfully buffer both partners against the downstream risk of depression sensitivity triggered by marital loneliness (Bulanda et al., 2024; Tucker et al., 2021).

## 5 Limitations

Although this study adds significantly to the body of literature, it is crucial to note some limitations. The study’s first restriction has to do with how the data was gathered. The self-reports of volunteers served as the basis for the data collection in this study. Thus, it is important to consider the social favorability effect as well as the potential for biased reactions. Furthermore, it should be noted that the study only assesses the study’s concepts within the context of the employed measurement instruments. In addition to the questionnaire, other measurement methods such as peer evaluation and observation can be helpful in subsequent research. Additionally, there is a restriction on the sample structure. The study only included married Turkish people, so it is important to exercise caution when extrapolating the results. Research involving diverse cultural groups is necessary to make the results applicable to a larger audience. Lastly, the study’s methodology has a limitation. Using a dyadic strategy, this study was carried out within the parameters of a cross-sectional design. The nature of the cross-sectional design precludes the establishment of causal correlations between variables. Therefore, it is advised that future research be done using a combination of dyadic and longitudinal approaches.

## 6 Implications

The findings of the present study offer several important implications, which can be categorized into methodological, clinical/therapeutic, and social dimensions.

From a methodological standpoint, the results underscore the critical necessity of employing dyadic analytical frameworks, such as the Actor-Partner Interdependence Mediation Model (APIMeM), when investigating psychological distress within marital contexts. Traditional individual-level analyses often fail to capture the mutual influence spouses exert on each other. By demonstrating significant partner effects, this study highlights that future research must treat the marital dyad, rather than the individual, as the primary unit of analysis to fully comprehend the transmission of emotional and cognitive vulnerabilities (Abaei and Martin, 2023). Furthermore, the reliance on item parceling for unidimensional constructs in complex dyadic structural equation modeling provides a robust methodological template for future researchers aiming to optimize parameter stability in similar couple-based studies (Little et al., 2002).

From a clinical perspective, these dyadic findings suggest that family and couple therapists must recognize the systemic transmission of psychological distress and shift away from an exclusively individualistic focus. Interventions should actively address the emotional contagion of loneliness and the resulting maladaptive systemic beliefs regarding the fragility of positive emotions. By helping couples dismantle the perception that happiness is fundamentally fleeting and by reconstructing secure, responsive relational bonds, therapeutic practices can successfully buffer both partners against the downstream risk of depression sensitivity triggered by marital loneliness (Bulanda et al., 2024). Furthermore, treating one partner’s depression sensitivity without addressing the spousal emotional climate and shared cognitive schemas may lead to incomplete recovery or high relapse rates.

On a broader social level, the findings emphasize that marital loneliness is a hidden yet profound public health risk. Social initiatives and community programs often target loneliness among unmarried or socially isolated individuals, overlooking the fact that individuals can experience severe emotional loneliness within a marriage (Rokach et al., 2022). Public health campaigns and psychoeducational programs should aim to destigmatize marital distress and raise societal awareness about the importance of maintaining high-quality, supportive intimate relationships. Fostering community environments that promote relational wellbeing can serve as a primary preventative measure against the escalation of depressive symptoms across the broader adult population (Holt-Lunstad et al., 2015).

## 7 Conclusion

This study concluded that the fragility of happiness mediates the relationship between marital loneliness and depression sensitivity. In other words, the fragility of happiness in both the individual and their spouse mediates the relationship between loneliness and depression sensitivity. The dyadic model study showed that loneliness can occur in close relationships and should be addressed individually and in the context of reciprocal relational dynamics. According to the results, a person’s experience with loneliness may result in a more fragile perception of happiness for both themselves and their spouse. The fragility of happiness in this situation increases sensitivity to depression. When the actor and partner impacts were examined jointly, it became clear that how people handle loneliness has an impact on their relationship dynamics as well as their psychological wellbeing.

## Data Availability

The raw data supporting the conclusions of this article will be made available by the authors, without undue reservation.
